# Efficacy of combined strategies of physical activity, diet and sleep disorders as treatment in patients with chronic shoulder pain. A systematic review

**DOI:** 10.3389/fphys.2023.1221807

**Published:** 2023-09-04

**Authors:** Dina Hamed Hamed, Filip Struyf, Leo Pruimboom, Santiago Navarro-Ledesma

**Affiliations:** ^1^ Department of Physiotherapy, Faculty of Health Sciences, University of Granada, Melilla, Spain; ^2^ Department of Rehabilitation Sciences and Physiotherapy, Faculty of Health Sciences, University of Antwerp, Melilla, Spain; ^3^ University Chair in Clinical Psychoneuroimmunology (University of Granada and PNI Europe), Melilla, Spain; ^4^ PNI Europe, The Hague, Netherlands

**Keywords:** physical activity, rehabilitation, chronic shoulder pain, healthy life style, sleep wake disorders

## Abstract

**Introduction:** The objective of this systematic review was to analyze the existing scientific evidence on the influence of dietary strategies, exercise, and sleep disorders on the symptomatology of patients with chronic shoulder pain, as well as to assess the methodological quality of the literature collected.

**Methods:** The selection criteria were as follows: we included randomized controlled clinical trials written in English that investigated the effects of such interventions in patients with chronic shoulder pain and excluded studies where pre-operative rehabilitation or rehabilitation combined with corticosteroid injections was performed. We searched six databases Pubmed, Cochrane Library, Web of Science, CINAHL, Sportdiscus and Scopus, using the keywords “shoulder pain,” “fasting,” “physical therapy modalities,” “rehabilitation,” “exercise,” “circadian clocks,” and “chronic pain” to select randomized controlled clinical trials conducted in humans and written in English. The last search was conducted on 24/01/2023. (PROSPERO:CRD42023379925).

**Results:** We used the tool proposed by the Cochrane Handbook to assess the risk of bias in the included studies of the 17 studies included, nine had a high risk of bias, two studies had an unclear risk of bias, and the remaining six studies had a low risk of bias. A total of 17 articles were selected, including 10 studies that showed a positive influences of exercise on chronic shoulder pain and five studies that showed a negative influence of sleep disorders on this patient profile. The remaining two articles analyzed the influence of nutritional strategies and metabolic problems in patients with chronic shoulder pain. The total sample size of the 17 included articles amounted to 9,991 individuals.

**Discussion:** Studies confirm that exercise generates a hypoalgesic effect that improves chronic shoulder pain, functionality, and quality of life. Although dietary strategies and sleep disorders are known to influence chronic shoulder pain, there is a lack of studies that conduct interventions on these problems to assess how chronic shoulder pain varies.

## 1 Introduction

Chronic pain is among the 10 most predominant diseases in the world (Sá et al., 2019), affecting 20% of adults (Luime et al., 2004) with prevalence increasing as the population ages (Luime et al., 2004; Pieters et al., 2022). There are many types of chronic pain, with chronic musculoskeletal pain being defined as pain that recurs for more than 3 months, causing significant impact on functionality and metabolic stress (Cuevas-Cervera et al., 2022). Chronic shoulder pain is one of the most common types of musculoskeletal pain, affecting 67% of the population and ranking as the second most frequent chronic musculoskeletal ailment (Luime et al., 2004). Rotator cuff involvement is the most common pathology associated with shoulder pain (Naunton et al., 2020). The shoulder is a complex joint with a high degree of mobility. As such, any resulting pain can be disabling for work or daily activities, with a significant impact on both the patient and society at the socio-economic level (Luime et al., 2004; Tekeoglu et al., 2013). Chronic shoulder pain accounts for 12% of all weekly patients seeking medical help (Tekeoglu et al., 2013).

The understanding of chronic shoulder pain has evolved from a biomedical perspective to a more comprehensive approach (Elma et al., 2020a). It is now widely recognized that lifestyle factors such as nutrition, lack of sleep, and exercise can have a significant impact on overall health and chronic conditions (Elma et al., 2020a; Perez-Montilla et al., 2022). As a result, chronic pain in general, and shoulder pain in particular, are approached from a multifactorial standpoint.

Current public health recommendations recognize exercise as a key factor in the prevention, management, and treatment of numerous chronic diseases (Egan and Zierath, 2013). Therefore, exercise is considered an effective intervention for addressing chronic pain in several shoulder disorders (Littlewood et al., 2015; Mertens et al., 2022). This is because training-induced adaptations generate changes in mitochondrial function and metabolic regulation. Acute exercise improves insulin sensitivity throughout the body for up to 48 h after the cessation of exercise (Egan and Zierath, 2013). The current literature has evaluated the ability of a combination of nutritional interventions and exercise to improve metabolic health biomarkers (Aird et al., 2018). Eating behavior and food intake are factors that could influence the onset, maintenance, and perception of chronic musculoskeletal pain (Elma et al., 2020a).

Systemic metabolic stress appears to promote low-grade inflammation and insulin resistance, which may be associated with disease risk (Burne et al., 2019). Dietary strategies to prevent metabolic syndrome are classified into 3 types: calorie restriction, intermittent fasting, and time-restricted eating (Moon et al., 2020). Current research shows that a high-fat diet has a direct impact on the immune system by causing increased cytokine levels, which directly impacts pain perception (Navarro-Ledesma et al., 2022a). Research is increasingly pointing to the existence of beneficial effects of fasting in chronic diseases (Perez-Montilla et al., 2022). Recently, interventions based on intermittent fasting have shown interest as an alternative to conventional dietary strategies to improve metabolic biomarkers in both healthy and clinical populations (Aird et al., 2018), demonstrating neurocognitive, physiological, and cellular benefits (Caron et al., 2022). Multiple studies have demonstrated that an intermittent diet produces changes in the microbial composition of the intestine and an increase in anti-inflammatory effects. Decreasing the anti-inflammatory effects improves insulin resistance and metabolic processes (Caron et al., 2022). The benefits of intermittent fasting are directly related to mechanisms associated with different states of chronic pain (Caron et al., 2022). The World Health Organization (WHO) also recognizes the importance of diet: “Nutrition is coming to the forefront as one of the main modifiable determinants of chronic disease effects, and scientific evidence increasingly supports the view that dietary alterations have strong effects (Elma et al., 2020a). Dietary factor patterns are suggested as an important indicator of chronic low-level systemic inflammation, which is a factor associated with chronic diseases (Tuttolomondo et al., 2019a). Thus, it is possible to predict a series of chronic diseases associated with unhealthy dietary behaviors or poor diet (Elma et al., 2020b).

There is increasing evidence of the relationship between chronic pain and sleep disturbances, with the use of drugs having a direct effect on sleep quality (Woo and Ratnayake, 2020). Sleep is a biological phenomenon necessary for body regulation and quality of life at any age (Pereira et al., 2020). The lack of sleep can negatively influence health, causing alterations in metabolic health and disorders in the endocrine system and immune pathway (Lee et al., 2017; Pereira et al., 2020). Specifically, irregular sleep patterns have been associated with adverse outcomes such as obesity and glucose metabolism (Lee et al., 2017). A systematic review confirms that the lack of sleep is a factor that can worsen pain, with those people who suffer from chronic pain being 18 times more likely to meet criteria for a clinical diagnosis of insomnia (Whibley et al., 2019). Studies show that at least 50% of patients with various types of chronic pain complain of significant sleep disorders (Smith and Haythornthwaite, 2004), with an estimated 50%–88% of these patients suffering from inadequate sleep (Palada et al., 2020). Chronic pain and insomnia are major health problems worldwide. In addition, the literature has shown that there is a bidirectional relationship between sequential measures of pain and sleep, with daytime pain being related to nighttime sleep deficit (Gao et al., 2020). The sleep-wake cycle is controlled by different hormones produced by the hypothalamus and circadian clock (Edwards et al., 2008).

Pain is also regulated by the circadian system (Palada et al., 2020), with circadian rhythms influencing almost every aspect of life (Segal et al., 2018). Virtually all organisms have circadian rhythms that help them adapt to the day-night relationship (Lucassen et al., 2016); they act as an adaptive mechanism to coordinate cellular processes, physiological functions and behaviors with the daily cycle of 24 h (Hood and Amir, 2017), which includes coordination of the immune system (Lucassen et al., 2016). Chronobiology is defined as the study of biological rhythms (Segal et al., 2018), with a focus on circadian rhythms, pain control and neuroimmune responses (Palada et al., 2020).

Circadian rhythms play a vital role in health, and prolonged clock alterations are associated with negative health consequences. As age increases, the circadian system undergoes significant changes that affect behavioral rhythms, temperature regulation, and hormone release (Edwards et al., 2008). Disruption of the circadian clock has been associated with decreased health conditions and may be present in metabolic syndromes, neurodegenerative diseases, chronic musculoskeletal pain, and inflammatory diseases (Segal et al., 2018; Navarro-Ledesma et al., 2022b). Therefore, disruption of circadian rhythms confirms the presence of mitochondrial dysfunction, which facilitates changes in the gut microbiota, immune function, and the autonomic nervous system (Segal et al., 2018).

There is an urgent need to better understand the effect of circadian rhythms on human development and maintenance (Whibley et al., 2019), but there is a great lack of studies in this regard. Therefore, it is of great relevance and interest to analyze the current literature on the interventions that have the greatest impact on biorhythm in patients with chronic musculoskeletal shoulder pain.

The objective of this literature review was to analyze the existing scientific evidence on the influence of dietary strategies, exercise and sleep disorders on the symptomatology of patients with chronic shoulder pain. In addition, to analyze the methodological quality of the literature collected.

## 2 Materials and methods

### 2.1 Study design

A systematic review was carried out following the recommendations of the Preferred Reporting Items for Systematic Review and Meta-Analysis (PRISMA) standard (Page et al., 2021), in which clinical studies have been included. The process was carried out using the PICOS strategy. The systematic review protocol was registered at the International Prospective Register of Systematic Reviews (PROSPERO: CRD42023379925). The aim of this search was to find scientific evidence on the influence of dietary strategies, exercise and sleep disorders on the pain suffered by patients with chronic shoulder pain.

### 2.2 Documentary sources consulted

The following computerized databases were consulted: Pubmed, Cochrane Library, Web of Science, CINAHL, Sportdiscus and Scopus.

### 2.3 Search strategy

To develop the search strategy, keywords extracted from the Medical Subject Headings (MesH) thesaurus were used: “shoulder pain,” “shoulder,” “tendinopathy,” “rotator cuff injuries,” “bursitis,” “synovitis,” “shoulder impingement syndrome,” “rotator cuff tear arthropathy,” “fasting,” “caloric restriction,” “diet,” “ketogenic,” “physical therapy modalities,” “rehabilitation,” “exercise,” “pain,” “chronic pain,” “healthy life style” and “quality of life/psychology,” “circadian clock,” “circadian rhythm,” “sleep wake disorders.” The following terms that were not obtained from the MesH thesaurus were also used: “chronic shoulder pain,” “tenosynovitis,” “tendinitis,” “tendinosis,” “adhesive capsulitis,” “frozen shoulder,” “rotator cuff” “calcinosis,” “shoulder arthritis,” “osteoporosis,” “nutritional strategies,” “physiotherapy,” “physical therapy,” “healthy,” “quality of life,” “psychological factors” and “functionality.” These terms were combined with the logical operators AND and OR. The terms had to appear in the title, abstract and keywords.

The last search was conducted on 24/01/2023.

Table 1 shows the search strategy performed in Pubmed. Annex 1 shows the rest of search strategies detailed in Supplementary Table S1.

**TABLE 1:** Search strategy.

Database	Search strategy
Pubmed	{[“Shoulder Pain”(Mesh) OR “Shoulder”(Mesh) OR “Tendinopathy”(Mesh) OR “Rotator Cuff Injuries”(Mesh) OR “Bursitis”(Mesh) OR “Synovitis”(Mesh) OR “Shoulder Impingement Syndrome”(Mesh) OR “Rotator Cuff Tear Arthropathy”(Mesh)] AND [“Circadian Clocks”(Mesh) OR “Circadian Rhythm”(Mesh) OR “Chronobiology “Disorders”(Mesh) OR “Chronobiology Discipline”(Mesh) OR “Sleep Wake Disorders”(Mesh) OR “insulin”(Mesh) OR “Fasting”(Mesh) OR “Caloric Restriction”(Mesh) OR “Diet, Ketogenic”(Mesh)] AND [“Pain”(Mesh) OR “Chronic Pain”(Mesh) OR “Healthy Lifestyle”(Mesh) OR “Quality of Life/psychology”(Mesh)]} OR {[“Shoulder pain”(tw) OR “shoulder”(tw) OR “Chronic shoulder pain”(tw) OR “Tendinopathy” (tw) OR “Tenosynovitis” (tw) OR “Tendinitis” (tw) OR “Tendinosis” (tw) OR “Bursitis” (tw) OR “Adhesive capsulitis” (tw) OR “Frozen shoulder” (tw) OR “Synovitis” (tw) OR “Rotator cuff” (tw) OR “Rotator Cuff Injuries”(tw) OR “Rotator Cuff Tear Arthropathy"(tw) OR “Shoulder impingement syndrome” (tw) OR “Shoulder Arthritis” (tw)] AND (“Circadian Clocks”(tw) OR “Circadian Rhythm”(tw) OR “Chronobiology “Disorders”(tw) OR “Chronobiology Discipline”(tw) OR “Sleep Wake Disorders”(tw) OR “insulin”(tw) OR “Fasting”(tw) OR “Caloric Restriction”(tw) OR “Diet, Ketogenic”(tw) OR “Nutritional strategies” (tw) OR “therapy” (tw)] AND [“Pain” (tw) OR “Chronic Pain”(tw) OR “Healthy”(tw) OR “Healthy Lifestyle”(tw) OR “Quality of life” (tw) OR “Quality of Life/psychology”(tw) OR “Psychological factors” (tw) OR “Funcionality” (tw)]}

### 2.4 Selection criteria

The selection process was carried out by two reviewers.

#### 2.4.1 Inclusion criteria

Only randomized controlled clinical trials that investigated the influence of nutritional strategies, exercise, and sleep disorders on the symptomatology of patients with chronic shoulder pain and were written in English were included in this review.

#### 2.4.2 Exclusion criteria

Studies where exercise is examined after or combined with corticosteroid injections.

Studies where patients undergo pre-operative rehabilitation.

### 2.5 Study selection process

Firstly, the Rayyan QCRl program (Ouzzani et al., 2016) was used to remove duplicate studies identified in the different databases. Then, two reviewers performed independently a preliminary screening by reading the title, followed by a more thorough screening by reading the title and abstract of the remaining studies. Finally, the full text of the articles that appeared to meet the inclusion criteria were read in detail. One reviewer was responsible for overseeing the decisions of the two reviewers.

### 2.6 Data extraction

The PICO strategy was used to extract data, including study characteristics (author, year of publication, type of study design, and study location), sample characteristics (size, age, and gender), intervention characteristics (type of intervention, nutritional strategies, sleep, or exercise) and main outcomes (assessment tools, follow-up, and intervention outcomes). Data were recorded in an Excel spreadsheet. Data were extracted by one researcher and checked by another, independently.

### 2.7 Risk of bias assessment tool

To assess the risk of bias in the included studies, the tool proposed by the Cochrane Manual of Systematic Reviews of Interventions (Higgins and Green, 2011) was used. This tool assesses 7 domains, where each domain is evaluated with three possibilities: “High risk”(−), “low risk”(+) and “unclear risk”(?). The domains that have been used for the risk of bias assessment are the following: selection bias, performance bias, detection bias, attrition bias, reporting biases and finally, other sources of bias where we could point out unaddressed biases that we consider important. The risk of bias was assessed by two reviewers independently.

### 2.8 Quality of the evidence

The tool used to assess the quality of evidence for the reported results in the studies is the Grading of Recommendations, Assessments, Development and Evaluation (GRADE) system (Aguayo-Albasini et al., 2014). This system defines the quality of evidence as the degree of confidence we have in the estimate of an effect to make a recommendation. The assessment of evidence quality includes factors such as the risk of bias in the study, inconsistency, imprecision, publication bias, indirect results, and other factors that may affect the quality of the evidence. The quality of evidence was assessed by two reviewers independently. The characterization of the evidentiary quality was delineated as follows (Sá et al., 2019): Elevated (subsequent investigations are improbable to substantially alter our assurance in the effect estimate, and there exists no recognized or suspected predisposition towards reporting biases: all realms are satisfied) (Luime et al., 2004). Intermediate (subsequent research is anticipated to wield a significant influence on our confidence in the effect estimate and has the potential to modify the estimate: one of the realms remains unfulfilled) (Pieters et al., 2022). Diminished (future inquiries are likely to exert a notable impact on our confidence in the effect estimate and are liable to amend the estimate: two of the realms are unmet) (Cuevas-Cervera et al., 2022). Extremely diminished (our certitude regarding the estimate is uncertain: three of the realms remain unmet) (Guyatt et al., 2008).

## 3 Results

### 3.1 Study identification and selection process

**FIGURE 1:**
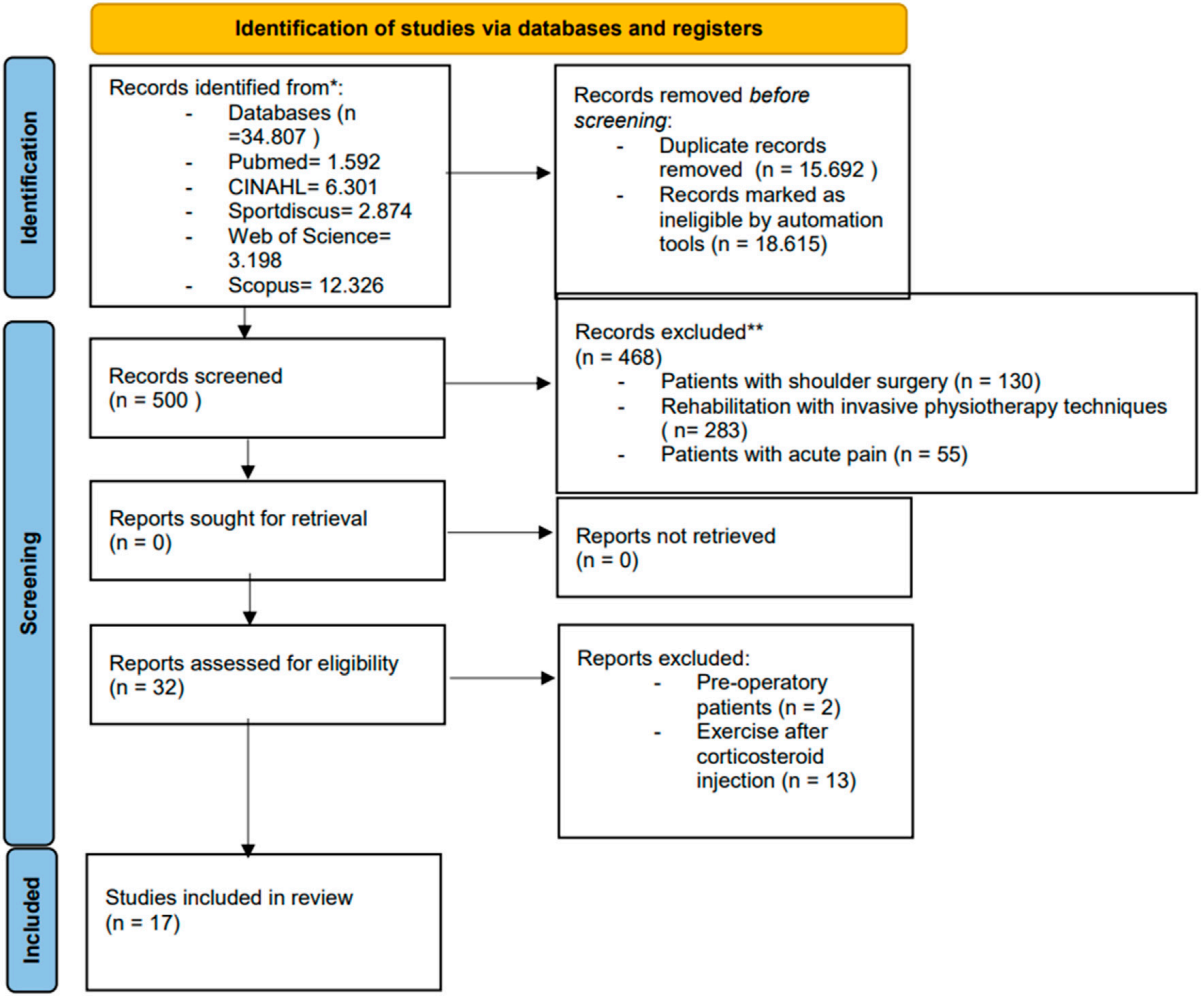
Flow diagram illustrating the study selection process.

In the process of identifying and selecting articles involved locating a total of 34,807 articles in various computerized databases. After eliminating duplicates, 19,115 articles were left, and their study titles were read. A title and abstract reading of 500 articles was carried out to determine whether the selected studies met the inclusion criteria. A total of 32 articles met these criteria and were evaluated in full text. After reading the full text, 17 studies (Lombardi et al., 2008; Rechardt et al., 2010; Østerås et al., 2010; Cho et al., 2013; Mulligan et al., 2015; Dilek et al., 2016; Abate et al., 2017; Turgut et al., 2017; Jeon and Chon, 2018; Khazzam et al., 2018; Vallés-Carrascosa et al., 2018; Toprak and Erden, 2019; Mohamed et al., 2020; Berg et al., 2021; Kamonseki et al., 2021; Alanazi et al., 2022; Hwang and Oh, 2022) were included in this literary review. The study selection process is illustrated in the flow diagram in Figure 1.

### 3.2 General characteristics of the selected studies

The studies included in this literature review comprised of 17 clinical trials (Lombardi et al., 2008; Rechardt et al., 2010; Østerås et al., 2010; Cho et al., 2013; Mulligan et al., 2015; Dilek et al., 2016; Abate et al., 2017; Turgut et al., 2017; Jeon and Chon, 2018; Khazzam et al., 2018; Vallés-Carrascosa et al., 2018; Toprak and Erden, 2019; Mohamed et al., 2020; Berg et al., 2021; Kamonseki et al., 2021; Alanazi et al., 2022; Hwang and Oh, 2022). The publication period ranged from 2008 (Lombardi et al., 2008) to 2022 (Yazmalar et al., 2016; Hwang and Oh, 2022), with the year 2017 (Abate et al., 2017; Turgut et al., 2017; Jeon and Chon, 2018; Vallés-Carrascosa et al., 2018) having the most published articles.

The total sample size of the 17 included articles amounted to 9,991 individuals. One study (Rechardt et al., 2010) has the largest population (*n* = 8,028), while another study (Berg et al., 2021) had the lowest number of participants (*n* = 21).

The sample sum size for the 2 included studies on nutritional strategies (Rechardt et al., 2010; Abate et al., 2017) comprises a total of 8,208 subjects (82.15%). The sample size sum for the 10 included studies on exercise (Lombardi et al., 2008; Rechardt et al., 2010; Østerås et al., 2010; Cho et al., 2013; Mulligan et al., 2015; Dilek et al., 2016; Abate et al., 2017; Turgut et al., 2017; Jeon and Chon, 2018; Khazzam et al., 2018; Vallés-Carrascosa et al., 2018; Toprak and Erden, 2019; Mohamed et al., 2020; Berg et al., 2021; Kamonseki et al., 2021; Alanazi et al., 2022; Hwang and Oh, 2022) is a total of 489 people (4.89%). The sample sum size for the 5 included studies on sleep disorders (Cho et al., 2013; Mulligan et al., 2015; Khazzam et al., 2018; Toprak and Erden, 2019; Hwang and Oh, 2022) is a total of 1,294 (12.95%).

The population in all clinical trials included both female and male subjects. The age range in all clinical trials is quite wide and spans from 18 years onwards. Mohamed et al. (2020) has the smallest age range, which is between 40 and 60 years of age.

There were no adverse effects related to the implementation of the interventions. The majority of dropouts in the experimental group sample were due to a lack of commitment by the patients to attend the sessions.

These characteristics can be seen in Table 2.

**TABLE 2:** General characteristics of the included studies and sample.

Author	Year	Country	Study design	Intervention	Initial sample	Average age (years ± SD)	Sex
Lombardi et al. (2008)	2008	Brasil	RCT	Exercise	*n* = 60	56.32 ± 11.6	Male and female
Østerås et al. (2010)	2010	Norway	RCT	Exercise	*n* = 61	46.1	Male and female
Rechardt et al. (2010)	2010	Finland	RCT	Nutrition	*n* = 8,028	52.9	Male and female
Cho et al. (2013)	2013	South Korea	RCT	Sleep	*n* = 190	54.8 ± 8.1	Male and female
Dilek et al. (2016)	2015	Republic of Turkey	RCT	Exercise	*n* = 61	50.06	Male and female
Mulligan et al. (2015)	2015	USA	RCT	Sleep	*n* = 343	66.0 ± 11.7	Male and female
Abate et al. (2017)	2017	Italy	RCT	Nutrition	*n* = 180	59.2 ± 7.1	Female
Vallés-Carrascosa et al. (2018)	2017	Spain	RCT	Exercise	*n* = 22	59	Male and female
Turgut et al. (2017)	2017	Republic of Turkey	RCT	Exercise	*n* = 36	39.5	Male and female
Jeon and Chon (2018)	2017	Korea	RCT	Exercise	*n* = 50	50.6 ± 6.82	Male and female
Toprak and Erden (2019)	2018	Republic of Turkey	RCT	Sleep	*n* = 148	59.32 ± 13.91	Male and female
Khazzam et al. (2018)	2018	USA	RCT	Sleep	*n* = 391	56.8	Male and female
Mohamed et al. (2020)	2020	Egypt	RCT	Exercise	*n* = 80	51.93 ± 6.16	Male and female
Berg et al. (2021)	2020	Norway	RCT	Exercise	*n* = 21	50 ± 14	Male and female
Kamonseki et al. (2021)	2021	Brasil	RCT	Exercise	*n* = 64	39	Male and female
Hwang and Oh (2022)	2022	Korea	RCT	Sleep	*n* = 222	49.5	Male and female
Alanazi et al. (2022)	2022	Saudi Arabia	RCT	Exercise	*n* = 34	39.1 ± 7.94	Male and female

Abbreviations: RCT, randomized controlled clinical trial; SD, standard deviation.

### 3.3 Risk of bias in the included studies

The risk of bias assessment showed that at least one domain had a high risk of bias in the included studies. The risk of bias varied depending on the type of intervention.

For studies on exercise interventions, the risk of bias seemed to be lower, with a low risk of bias in all domains. The studies by Turgut et al. (2017); Jeon and Chon (2018); Mohamed et al. (2020); Berg et al. (2021); Kamonseki et al. (2021); Alanazi et al. (2022) had the lowest risk of bias. In contrast, the studies by Rechardt et al. (2010); Cho et al. (2013); Dilek et al. (2016); Mulligan et al. (2015); Abate et al. (2017) and Vallés-Carrascosa et al. (2018) had the highest risk of bias.

For studies on nutritional strategies and sleep disorders, all articles had a high risk of bias in almost all domains, with reporting bias being the only domain with a low risk of bias. It was difficult to obtain a low risk of bias in these studies as no intervention was performed.

Table 3 presents the risk of bias for the included articles. Different colors indicate the methodological quality of the studies: high risk (red), unclear risk (yellow), and low risk of bias (green).

**TABLE 3:** Risk of bias in the included studies.

	Lombardi et al. (Lombardi et al., 2008)	Osteras et al. (Østerås et al., 2010)	Rechardt et al. (Rechardt et al., 2010)	Cho et al. (Cho et al., 2013)	Dilek et al. (Dilek et al., 2016)	Mulligan et al. (Mulligan et al., 2015)	Abate et al. (Abate et al., 2017)	Carrascosa et al. (Vallés-Carrascosa et al., 2018)	Turgut et al. (Turgut et al., 2017)	Young jeon et al. (Jeon and Chon, 2018)	Toprak et al. (Toprak and Erden, 2019)	Khazzam et al. (Khazzam et al., 2018)	Mohamed et al. (Mohamed et al., 2020)	Berg K.O et al. (Berg et al., 2021)	Kamonseki et al. (Kamonseki et al., 2021)	Hwang et al. (Hwang and Oh, 2022)	Allanazi A et al. (Alanazi et al., 2022)
Selection bias	+	+	?	?	?	?	?	+	+	+	?	?	+	+	+	?	+
Selection bias	+	+	?	?	?	?	?	?	+	+	?	?	+	+	+	?	+
Implementation bias	?	+	?	?	?	?	?	?	+	+	?	?	+	+	+	?	+
Detection bias	+	-	?	?	?	?	?	?	+	+	?	?	+	+	+	?	+
Wear bias	?	-	-	?	?	?	?	?	+	+	?	?	+	+	+	?	+
Notification bias	-	+	+	+	+	+	+	+	+	+	+	+	+	+	+	?	+
Other sources of bias	+	+	+	+	+	+	+	+	+	+	+	?	+	+	+	+	+

Abbreviations: (+), Low bias risk; (−), High bias risk; (?), Unknown bias risk.

### 3.4 Intervention characteristics

All the studies included in this narrative review analyze factors that influence the body’s biorhythm.

Three types of strategies that affect the biorhythm were investigated: physical exercise, which was evaluated in 10 studies (Lombardi et al., 2008; Rechardt et al., 2010; Østerås et al., 2010; Cho et al., 2013; Mulligan et al., 2015; Dilek et al., 2016; Abate et al., 2017; Turgut et al., 2017; Jeon and Chon, 2018; Khazzam et al., 2018; Vallés-Carrascosa et al., 2018; Toprak and Erden, 2019; Mohamed et al., 2020; Berg et al., 2021; Kamonseki et al., 2021; Alanazi et al., 2022; Hwang and Oh, 2022), nutritional strategies, which were examined in 2 studies (Rechardt et al., 2010; Abate et al., 2017), and sleep disorders, which were analyzed in 5 articles (Cho et al., 2013; Mulligan et al., 2015; Khazzam et al., 2018; Toprak and Erden, 2019; Hwang and Oh, 2022). In all these trials, chronic pain was the variable studied.

The characteristics of the studies evaluating physical exercise, nutritional strategies, and sleep as influential factors in pain were as follows:

#### 3.4.1 Physical exercise

1) Lombardi et al. (2008): A randomized study consisting of two treatment groups was conducted. An experimental group received a shoulder strengthening program that was performed twice a week for 2 months, and a control group did not receive any intervention.
2) Østerås et al. (2010): This study investigated the type and amount of exercise needed to improve symptoms in people with chronic subacromial pain. Two groups were established: one group received exercises prescribed by a physician, and another group performed conventional exercises. Pain was measured with the visual analogue scale (VAS) and the shoulder assessment questionnaire, with measurements taken at baseline, the end of the treatment, and 6- and 12-month follow-ups.
3) Dilek et al. (2016): This study explored the effectiveness of proprioceptive exercises in patients with subacromial impingement syndrome. Two groups were established, a control group received only physiotherapy sessions, while an experimental group received both physiotherapy and exercises to improve pain. Pain was measured using the VAS, taking into account both active pain and night pain perceived by the patients at rest.
4) Vallés-Carrascosa et al. (2018): The intervention in this study was a training program that was carried out for 4 weeks and was organized into 5 weekly training sessions. There were two groups, an experimental painful group where patients suffering from chronic shoulder pain performed the exercise program and an experimental non-painful group where asymptomatic patients performed the same exercise program. The intensity of shoulder pain was recorded before the start of the intervention, daily and after the program ended. A goniometer was used to assess the range of motion of the glenohumeral joint.
5) Turgut et al. (2017): Two groups were formed in this study. Each group ran a different exercise program in order to discover which exercise program turned out to be more effective. The first group performed strengthening and stretching exercises of the shoulder girdle and scapular stabilization exercises that focused on the kinetic chains, while the second group only performed stretches and exercises to strengthen the shoulder girdle. Pain was assessed before and after the interventions and in addition at the 6- and 12-week post intervention point.
6) Jeon and Chon (2018): In this study, stabilization exercises of the glenohumeral and scapular joint are performed, all exercises were repeated for a total of 3 sets with 15 repetitions in each set There is an experimental group and a control group. Measurements of the variables were made before and after the intervention. Pain intensity was measured using the NRPS scale. The indepen dent t−test was used to assess the mean differences in the general characteristics of the subjects between the groups
7) Mohamed et al. (2020): This study consisted of an experimental group where the ability of a dynamic scapular exercise to improve mobility and shoulder pain was implemented, while the control group received a placebo program of exercises focused on the healthy joint. The shoulder pain and disability index (SPADI) was used to measure shoulder pain and disability.
8) Berg et al. (2021): This study aimed to determine whether the addition of high-intensity rotator cuff aerobic training to usual care could improve pain. Two groups were established, an experimental group where high-intensity exercises were performed in addition to usual care and a control group that only received their usual treatment. The intervention lasted 8 weeks and pain and disability were assessed using the SPADI index.
9) Kamonseki et al. (2021): This study performed a scapula focused exercise intervention for the improvement of chronic shoulder pain. Two groups were organized with group one performing scapular movements while group two performed stretching and strengthening exercises. The intervention lasted 8 weeks and the trainings were organized twice a week.
10) Alanazi et al. (2022): This study explores the effects of strengthening exercises on improving pain in patients with subacromial impingement syndrome. Two groups were formed, a control group where patients received ultrasound therapy, ice and stretching exercises and an experimental group where patients received the same program as the control group but with the addition of strengthening exercises. It was an 8-week program that was organized twice a week.

#### 3.4.2 Nutritional interventions

Rechardt et al. (2010): All participants completed an interview on musculoskeletal clinical symptoms and had a physical examination by a physician. Information was collected on demographic factors, lifestyle and factors related to physical loads. Participants donated fasting blood samples for the analysis of serum glucose, insulin, high-denensity lipoprotein cholesterol, triglycerides and C- reactive protein. Logistic regression models were performed using statistical analysis to study the determinants of pain in the shoulder joint.

Abate et al. (2017): Patients with tendinopathy, deltoid subacromial bursitis, calcified tendinopathy and adhesive tendinitis were included. Both shoulders were evaluated by ultrasound and age, sex, smoking, heavy work, hypertension, diabetes and hypercholesterolemia of the included patients were taken into account. Ultrasound, demographic, anthropometric and clinical characteristics of patients with unilateral and bilateral shoulder tears were compared. The χ2 test was used to evaluate associations between variables.

#### 3.4.3 Sleep interventions

Cho et al. (2013): Participants with chronic shoulder pain were assessed using the VAS, the American Shoulder and Elbow Surgeons Scale, the Korean Shoulder Scale, the Anxiety and Depression Scale and the Pittsburgh Sleep Quality Index (a questionnaire with 19 questions that obtains patient information about sleep habits during the previous month to measure subjective sleep quality; scoring subjective sleep quality, sleep attention, sleep duration, sleep disturbances, sleep medication use, and daytime dysfunction). A Pearson’s correlation analysis was used to assess relationships between scale scores and also a logistic regression analysis was performed to assess depression, anxiety and sleep disorders modified by shoulder pain. A statistical analysis was performed to investigate the relationship between sleep quality and shoulder pain.

Mulligan et al. (2015): Sleep quality was assessed using the Pittsburg Sleep Quality Questionnaire. (PSQI). A one-way analysis variance was performed, with the help of the SigmaPlot software version 12.5, to analyze the between groups measurement difference.

Toprak and Erden (2019): The PSQI questionnaire was used to evaluate sleep disorders. A statistical analysis was performed to determine the relationship between the variables.

Khazzam et al. (2018): The participants completed an 8-page questionnaire on shoulder symptoms and sleep disorders, used the VAS for shoulder pain and the Pittsburgh sleep quality index for sleep disorders (this questionnaire consists of 19 questions divided into seven subcategories: sleep quality, sleep duration latency and disturbances, usual sleep efficiency, use of sleep medication and daytime dysfunction). All student test data were analyzed using the SigmaPlot version 12.5 computer program.

Hwang and Oh (2022): The SPADI scale was used to assess pain index and shoulder disability. Sleep quality was assessed using a 4-point scale.

Table 4 shows the characteristics of the intervention in detail.

**TABLE 4:** Sample characteristics.

Author	Year	Intervention		Groups	Length of intervention (weeks)	Variables	Instrument of measurement
Lombardi et al. (Lombardi et al., 2008)	2008	Exercise	GC: 25	GE:30	2	Pain	Likert scale
Osteras et al. (Østerås et al., 2010)	2010	Exercise	G1:31	G2:30	12	Pain	VAS
Rechardt et al. (Rechardt et al., 2010)	2010	Nutrition	-	-	32	Pain	Health survey 2000
Cho et al. (Cho et al., 2013)	2013	Sleep	GC:60	GE:130	-	Pain	VAS
Dilek et al. (Dilek et al., 2016)	2015	Exercise	GC:30	GE:31	12	Pain	VAS
Mulligan et al. (Mulligan et al., 2015)	2015	Sleep				Pain	VAS
Abate et al. (Abate et al., 2017)	2017	Nutrition				Pain	
Vallés-Carrascosa et al. (Vallés-Carrascosa et al., 2018)	2017	Exercise	GC SIN DOLOR:11	GE CON DOLOR:11	4	Pain	VAS
Turgur et al. (Turgut et al., 2017)	2017	Exercise	GC:15	GE:15	2	Pain	SPADI
Na-Young Jeon et al. (Jeon and Chon, 2018)	2017	Exercise	GC:20	GE:20	?	Pain	NRPS
Toprak et al. (Toprak and Erden, 2019)	2018	Sleep	GC:72	GE:76	-	Pain	NRPS
Khazzam et al. (Khazzam et al., 2018)	2018	Sleep				Pain	VAS
Mohamed et al. (Mohamed et al., 2020)	2020	Exercise	G1:33	G2:33	8	Pain	SPADI
Berg K.O et al. (Berg et al., 2021)	2020	Exercise	GE:13	GC:8	8	Pain	SPADI
Kamonseki et al. (Kamonseki et al., 2021)	2021	Exercise	G1:32	G2:32	8	Pain	NRPS
Hwang et al. (Hwang and Oh, 2022)	2022	Sleep				Pain	SPADI
Alanazi A et al. (Alanazi et al., 2022)	2022	Exercise	GE:18	GC:16	8	Pain	VAS

Abbreviations: Control group, (CG); experimental group, (EG); Group 1, (G1); Group 2, (G2); Visual Analogue Scale, (VAS); Shoulder Pain and Disability Index, (SPADI); Numeric Pain Rating Scale, (NPRS).

### 3.5 Results

#### 3.5.1 Physical exercise

Lombardi et al. (2008): An improvement in pain was observed in the experimental group compared to the control group.

Østerås et al. (2010): In subjects with long-term subacromial pain syndrome, medical exercise therapy is superior to a conventional exercise program.

Cho et al. (2013): The intervention group showed significant improvement in pain compared to the control group, but at 12 weeks no significant improvements were found between groups (i.e., acute effect but no long-term effect).

Vallés-Carrascosa et al. (2018): All participants completed the program without adverse reactions. The pre-test and post-test differences between both groups showed that both groups improved similarly in all dependent variables, without significant differences between groups.

Turgut et al. (2017): The groups showed improvements in pain scores with no differences between the groups. Progressive exercise training independent of scapula stabilization exercises decreases pain intensity in patients with subacromial impingement syndrome.

Jeon and Chon (2018): In the experimental group there was a significant improvement in shoulder stabilization and pain intensity compared to the control group.

Mohamed et al. (2020): The results of the study showed that after 2 and 6 months there were significant differences between the experimental group and the control group in pain improvement on the SPADI scale.

Berg et al. (2021): In the experimental group, the SPADI score for pain was reduced by 22 points and the change from pre-test to post-test was significant when compared to the control group. Patients were re-evaluated by ultrasound, which showed an increase in tendon blood in the experimental group, so high-intensity rotator cuff training proved to be a feasible tool.

Kamonseki et al. (2021): Scapular movement training reduces pain compared to stretching and strengthening exercises.

Alanazi et al. (2022): The experimental group showed an improvement over time in shoulder symptomatology compared to the control group.

#### 3.5.2 Nutritional interventions

Rechardt et al. (2010): Statistical analyses showed that age, education, BMI, waist-hip circumference, metabolic syndrome, diabetes, and physical load were associated with joint pain in men and women. Additionally, smoking was associated to males and insulin resistance to women. There is a strong relationship between shoulder pain and all factors related to weight, especially abdominal obesity. An association was also found between type 1 diabetes mellitus and chronic rotator cuff tendinitis.

Abate et al. (2017): It was observed that older age, heavy repetitive work, and diabetes were significantly prevalent in patients with bilateral tears: however, no statistical significance was found for hypertension, hypercholesterolemia or smoking. In regards to these statistical data, it is concluded that metabolic risk factors are more involved in the presence of shoulder tendon degeneration.

#### 3.5.3 Sleep interventions

Cho et al. (2013): A positive relationship between sleep disturbances and the duration of symptoms was observed in people with chronic shoulder pain. Additionally, a correlation between pain scores and scores on the sleep scale was observed.

Mulligan et al. (2015): This study shows that patients with subacromial impingement syndrome, rotator cuff tears, glenohumeral osteoarthritis and shoulder adhesive capsulitis suffer from high sleep quality index scores.

Toprak and Erden (2019): A close relationship was found between pain, anxiety, sleep disturbances and quality of life.

Khazzam et al. (2018): Factors such as sleep, depression, diabetes mellitus and a high BMI correlate with poorer sleep quality, therefore these factors influence night-time shoulder pain.

Hwang and Oh (2022): The effects of sleep disorders and depression are noted to be significant in terms of shoulder pain.

### 3.6 Quality of the evidence

The quality of evidence in this systematic review is low. Assessments have heavily relied on the risk of bias of the trials and the imprecision of their results, primarily due to the disparity in sample sizes.

However, the quality of evidence is higher in studies that use exercise as a method to improve pain (Lombardi et al., 2008; Rechardt et al., 2010; Østerås et al., 2010; Cho et al., 2013; Mulligan et al., 2015; Dilek et al., 2016; Abate et al., 2017; Turgut et al., 2017; Jeon and Chon, 2018; Khazzam et al., 2018; Vallés-Carrascosa et al., 2018; Toprak and Erden, 2019; Mohamed et al., 2020; Berg et al., 2021; Kamonseki et al., 2021; Alanazi et al., 2022; Hwang and Oh, 2022) since the risk of bias in these studies is high.

Conversely, studies on sleep disorders (Cho et al., 2013; Mulligan et al., 2015; Khazzam et al., 2018; Toprak and Erden, 2019; Hwang and Oh, 2022) and dietary strategies (Rechardt et al., 2010; Abate et al., 2017) have low-quality of evidence concerning pain improvement since the risk of bias in the reported studies is low.

For more detailed information, see Supplementary Table S2 in Annex 5.2.

## 4 Discussion

The objective of this systematic review was to analyze the existing scientific evidence on the influence of dietary strategies, exercise, and sleep disorders on the symptomatology of patients with chronic shoulder pain. Additionally, we aimed to analyze the methodological quality of the collected literature.

The search strategies resulted in the selection of 17 clinical trials (Lombardi et al., 2008; Rechardt et al., 2010; Østerås et al., 2010; Cho et al., 2013; Mulligan et al., 2015; Dilek et al., 2016; Abate et al., 2017; Turgut et al., 2017; Jeon and Chon, 2018; Khazzam et al., 2018; Vallés-Carrascosa et al., 2018; Toprak and Erden, 2019; Mohamed et al., 2020; Berg et al., 2021; Kamonseki et al., 2021; Alanazi et al., 2022; Hwang and Oh, 2022). Among these studies, ten used exercise as a treatment for chronic shoulder pain (Lombardi et al., 2008; Rechardt et al., 2010; Østerås et al., 2010; Cho et al., 2013; Mulligan et al., 2015; Dilek et al., 2016; Abate et al., 2017; Turgut et al., 2017; Jeon and Chon, 2018; Khazzam et al., 2018; Vallés-Carrascosa et al., 2018; Toprak and Erden, 2019; Mohamed et al., 2020; Berg et al., 2021; Kamonseki et al., 2021; Alanazi et al., 2022; Hwang and Oh, 2022), and the VAS, NPRS, LIKERT, and SPADI scales were the main tools used to measure pain. Two studies focused on the influence of nutritional disorders on chronic shoulder pain (Rechardt et al., 2010; Abate et al., 2017), and the remaining 5 studies investigated sleep disorders in chronic shoulder pain (Cho et al., 2013; Mulligan et al., 2015; Khazzam et al., 2018; Toprak and Erden, 2019; Hwang and Oh, 2022). Pain was assessed using the VAS, NPRS, and SPADI scales in these studies.

### 4.1 Chronic shoulder pain and exercise

The exercise group in each study showed significant improvements in terms of pain, and consequently, the quality of life of these patients also improved (Lombardi et al., 2008; Rechardt et al., 2010; Østerås et al., 2010; Cho et al., 2013; Mulligan et al., 2015; Dilek et al., 2016; Abate et al., 2017; Turgut et al., 2017; Jeon and Chon, 2018; Khazzam et al., 2018; Vallés-Carrascosa et al., 2018; Toprak and Erden, 2019; Mohamed et al., 2020; Berg et al., 2021; Kamonseki et al., 2021; Alanazi et al., 2022; Hwang and Oh, 2022). These results are consistent with the study by Yazmalar et al. (2016), which states that exercise has beneficial results on pain, depression, anxiety, disability, sleep, and quality of life for patients with chronic shoulder pain due to subacromial impingement syndrome (Yazmalar et al., 2016). A literature review highlighted a controlled study in which the authors state that aerobic exercise reduces pain sensitization in people suffering from chronic musculoskeletal pain due to the production of a hypoanalgesic effect. They also suggest that this may be due to aerobic exercise releasing endogenous opioids and beta-endorphins that cause hypoalgesia. It has also been proposed that it can activate nociceptive inhibitory mechanisms that reduce pain sensitivity (Tan et al., 2022). However, a meta-analysis of isometric exercise studies did not demonstrate any hypoanalgesic effect in people with chronic musculoskeletal pain (Wewege and Jones, 2021). These results are supported by another systematic review where hypoanalgesic effects were only found when isometric exercises focused on the quadriceps, were carried out in people with chronic musculoskeletal pain, although adjusting the weight, duration, and intensity of the exercise would be necessary to generate hypoalgesia (Bonello et al., 2021).

### 4.2 Chronic shoulder pain and nutritional strategies

It was observed that problems related to poor diet directly influence chronic pain (Rechardt et al., 2010; Abate et al., 2017). Although there are no studies that use dietary strategies as a treatment to relieve chronic shoulder pain symptoms, (Pietrzak, 2016) reflected on the association between patients with adhesive capsulitis, metabolic syndrome and chronic low-grade inflammation. These pathophysiological mechanisms are likely perpetuated by the positive regulation in the production of proinflammatory cytokines (Pietrzak, 2016). The review by Cuevas-Cervera et al. (2022) verifies the use of nutritional strategies, especially the use of intermittent fasting, to improve inflammation and oxidative stress conditions in chronic pain, which helps improve the patient’s quality of life (Cuevas-Cervera et al., 2022). These results coincide with a literature review confirming that there are diets with antioxidant and anti-inflammatory components capable of influencing peoples’ chronic pain (Henderson, 2005). In a study published in 2018, a plant-based diet was observed to improve pain in people with chronic musculoskeletal pain, demonstrating that nutritional interventions combined with physiotherapy treatments can positively influence chronic musculoskeletal pain (Towery et al., 2018).

### 4.3 Chronic shoulder pain and sleep disorders

The literature review revealed that chronic shoulder pain was linked sleep disorders in 5 of the selected studies (Cho et al., 2013; Mulligan et al., 2015; Khazzam et al., 2018; Toprak and Erden, 2019; Hwang and Oh, 2022); however, no treatment strategies were proposed to alleviate pain and improve sleep these patients. These findings are consistent with the studies by Schuh-Hofer et al. (2013) and Bascour-Sandoval et al. (2021) where the authors noted the high prevalence of sleep disorders in people with chronic pain. The quality and quantity of sleep are important biological resources for regulating pain homeostasis processes (Haack et al., 2007a). According to one study (Haack et al., 2007b), insufficient sleep can promote pain by increasing IL-6 levels, and sleep disorders are commonly associated with increased inflammation (Haack et al., 2007b). Vgontzas et al. (2004) also found that sleep loss leads to an increase in proinflammatory cytokines (Vgontzas et al., 2004). Additionally, the study by Hallman and Lyskov (2012) showed that circadian rhythm disturbances, primarily a decrease in sympathetic nocturnal activity, are linked to chronic neck and shoulder pain, with nervous system hyperactivity being a significant factor in the development and persistance of chronic muscle pain (Hallman and Lyskov, 2012). A lack of sleep, which is prevalent among the general population, is also associated with metabolic and endocrine variations that may have long-term pathophysiological consequences (Leproult and Van Cauter, 1999).

Lastly, psychosocial factors, which can act as mediators between physical variations and perceived disability are other elements that should be taken into account when treating a patient suffering from chronic pain (Wolfensberger et al., 2016). Martinez-Calderón et al. associated psychological distress with disability in patients with chronic shoulder pain (Martinez-Calderon et al., 2017), while in another review by the same authors, a relationship between anguish syndromes, depressive syndromes, anxiety, worries and pain somatization, was found with high levels of pain intensity and disability in the same population (Martinez-Calderon et al., 2018).

### 4.4 Strengths and weaknesses of the study

This review highlights the insufficient scientific evidence on treatments that improve chronic shoulder pain through nutritional strategies and sleep improvements. Therefore, it proposes a comprehensive intervention that addresses chronic shoulder pain through exercise, nutritional strategies, and sleep since all these factors influence the biorhythm. However, certain limitations weaken the findings of this literary review, such as the lack of randomized controlled trials that demonstrate variations in chronic shoulder pain with a food or sleep intervention. Furthermore, grey literature was not included in the search, thus it may cause some impact on the results obtained. Finally, out of the 17 studies, nine had a high risk of bias, and this should also be taken into account when interpreting the results.

#### 4.4.1 Prospective

After reviewing and analyzing the results, it can be concluded that there is a lack of scientific evidence on a comprehensive treatment for chronic shoulder pain. Therefore, the following lines of research are proposed:

- The implementation of RCTs to evaluate the effectiveness of dietary strategies in improving chronic shoulder pain.
- The implementation of RCTs to evaluate the effectiveness of interventions that address sleep disorders and subsequently improve chronic shoulder pain.
- The implementation of RCTs that combine these strategies into a comprehensive treatment.
- To perform a quantitative data synthesis through a meta-analysis to evaluate which type of intervention had a greater influence on patients’ chronic shoulder pain.

Although the studies on exercise interventions showed the risk of bias to be lower, future research should consider the type of exercise and the optimal time of day for performing it, as well as a specific diet to enhance treatment effectiveness. On the other hand, the studies on nutritional strategies and sleep disorders showed a high risk of bias in almost all domains, so its conclusions must be interpreted with caution, and future studies with higher methodological quality are needed. In this regard, nutritional strategies for improving chronic pain have been recently proposed, as well as innovative proposals through fermented food, phytomelatonin, and mito-hormetic strategies (cold and heat exposure, breathing techniques) based on mitochondrial metabolism improvement (Tuttolomondo et al., 2019b; Cuevas-Cervera et al., 2022; Casanova et al., 2023).

## 5 Conclusion

Studies confirm that exercise generates a hypo analgesic effect that improves chronic shoulder pain, functionality and quality of life. It has been confirmed that chronic shoulder pain is influenced by dietary strategies and sleep disorders, but studies that carry out interventions on these problems to assess chronic shoulder pain variation are lacking, specifically in terms of methodological quality. Thus, more research following rigorous methodological criteria is necessary to corroborate the results of this study.

## Data Availability

The original contributions presented in the study are included in the article/Supplementary Materials, further inquiries can be directed to the corresponding author: snl@ugr.es.

## Supplementary material

SUPPLEMENTARY TABLE S1 Search strategies.

SUPPLEMENTARY TABLE S2 Grade system.
