# Supplementary material for: Efficacy of combined strategies of physical activity, diet and sleep disorders as treatment in patients with chronic shoulder pain. A systematic review
Source: Front Physiol. 2023 Sep 4;14:1221807. doi: 10.3389/fphys.2023.1221807 (PMC10507353; doi:10.3389/fphys.2023.1221807)

**Supplementary Material:**

**Supplementary table 1: Search Strategies**

| Database | Search Strategies |
| --- | --- |
| LILACS E IBECS | Tw:(("Shoulder pain” OR "shoulder" OR “Chronic shoulder pain” OR “Tendinopathy” OR “Tenosynovitis” OR “Tendinitis” OR “Tendinosis” OR “Bursitis” OR “Adhesive capsulitis” OR “Frozen shoulder” OR “Synovitis” OR “Rotator cuff” OR "Rotator Cuff Injuries" OR "Rotator Cuff Tear Arthropathy" OR “Shoulder impingement syndrome” OR “Shoulder Arthritis”) AND ("Circadian Clocks" OR "Circadian Rhythm" OR “Chronobiology Discipline" OR "Sleep Wake Disorders" OR "insulin" OR "Fasting" OR "Caloric Restriction" OR "Diet, Ketogenic" OR “Nutritional strategies” OR “Therapy”) AND ("Pain" OR "Chronic Pain" OR "Healthy" OR "Healthy Lifestyle" OR “Quality of life” OR "Quality of Life/psychology" OR “Psychological factors” OR “Funcionality” )) OR mh: (("Shoulder Pain"OR "Shoulder" OR "Tendinopathy" OR "Rotator Cuff Injuries" OR "Bursitis" OR "Synovitis" OR "Shoulder Impingement Syndrome" OR "Rotator Cuff Tear Arthropathy") AND ("Circadian Clocks" OR "Circadian Rhythm" OR "Chronobiology Discipline" OR "Sleep Wake Disorders" OR "insulin" OR "Fasting" OR "Caloric Restriction" OR "Diet, Ketogenic") AND ("Pain" OR "Chronic Pain" OR "Healthy Lifestyle" OR "Quality of Life/psychology")) |
| Cochrane Library | ID Search Hits  #1 (fasting) (Word variations have been searched) 56192  #2 (caloric restriction) (Word variations have been searched) 2974  #3 (ketogenic diet) (Word variations have been searched) 647  #4 (nutritional strategies) (Word variations have been searched) 6770  #5 (pain) (Word variations have been searched) 224233  #6 (chronic pain) (Word variations have been searched) 36446  #7 (healthy) (Word variations have been searched) 166983  #8 (healthy lifestyle) (Word variations have been searched) 6819  #9 (quality of life) (Word variations have been searched) 149792  #10 (quality of life psychology) (Word variations have been searched) 24910  #11 (psichological factors) (Word variations have been searched) 1  #12 (funcionality) (Word variations have been searched) 668  #13 MeSH descriptor: [Fasting] explode all trees 3507  #14 MeSH descriptor: [Caloric Restriction] explode all trees 955  #15 MeSH descriptor: [Diet, Ketogenic] explode all trees 115  #16 MeSH descriptor: [Pain] explode all trees 56266  #17 MeSH descriptor: [Chronic Pain] explode all trees 3180  #18 MeSH descriptor: [Healthy Lifestyle] explode all trees 1028  #19 "Chronobiology" 450  #20 "Sleep Wake Disorders" 1953  #21 insulin 68904  #22 MeSH descriptor: [Insulins] explode all trees 15403  #23 chronic pain 35847  #24 MeSH descriptor: [Chronic Pain] explode all trees 3180  #25 Therapy 787025  #26 circadian clocks 88  #27 circadian rhythm 5326  #28 chronobiology discipline 1  #29 shoulder pain 8869  #30 shoulder 14665  #31 chronic shoulder pain 1404  #32 tendinopathy 1302  #33 tenosynovitis 334  #34 tendinitis 1027  #35 tendinosis 149  #36 bursitis 689  #37 adhesive capsulitis 539  #38 frozen shoulder 662  #39 synovitis 1210  #40 rotator cuff 2393  #41 rotator cuff injuries 785  #42 rotator cuff tear arthropathy 53  #43 shoulder impingement syndrome 866  #44 shoulder arthritis 544  #45 MeSH descriptor: [Shoulder Pain] explode all trees 1138  #46 MeSH descriptor: [Shoulder] explode all trees 675  #47 MeSH descriptor: [Tendinopathy] explode all trees 1273  #48 MeSH descriptor: [Rotator Cuff Injuries] explode all trees 627  #49 MeSH descriptor: [Bursitis] explode all trees 477  #50 MeSH descriptor: [Synovitis] explode all trees 203  #51 MeSH descriptor: [Shoulder Impingement Syndrome] explode all trees 421  #52 MeSH descriptor: [Rotator Cuff Tear Arthropathy] explode all trees 15  #53 MeSH descriptor: [Circadian Clocks] explode all trees 22  #54 MeSH descriptor: [Circadian Rhythm] explode all trees 3123  #55 ((#29 OR #30 OR #31 OR #32 OR #33 OR #34 OR #35 OR #36 OR #37 OR #38 OR #39 OR #40 OR #41 OR #42 OR #43 OR #44) AND (#26 OR #27 OR #28 OR #29 OR #20 OR #21 OR #1 OR #2 OR #3 OR #4) AND (#5 OR #6 OR #7 OR #8 OR #9 OR #10 OR #11 OR #12)) OR ((#45 OR #46 OR #47 OR #48 OR #49 OR #50 OR #51 OR #52) AND (#53 OR #54 OR #13 OR #14 OR #15 OR #22) AND (#16 OR #17 OR #18)) 8983 |
| EBSCO | (("Shoulder pain” OR "shoulder" OR “Chronic shoulder pain” OR “Tendinopathy” OR “Tenosynovitis” OR “Tendinitis” OR “Tendinosis” OR “Bursitis” OR “Adhesive capsulitis” OR “Frozen shoulder” OR “Synovitis” OR “Rotator cuff” OR "Rotator Cuff Injuries" OR "Rotator Cuff Tear Arthropathy" OR “Shoulder impingement syndrome” OR “Shoulder Arthritis”) AND ("Circadian Clocks" OR "Circadian Rhythm" OR “Chronobiology Discipline" OR "Sleep Wake Disorders" OR "insulin" OR "Fasting" OR "Caloric Restriction" OR "Diet, Ketogenic" OR “Nutritional strategies” OR “Therapy”) AND ("Pain" OR "Chronic Pain" OR "Healthy" OR "Healthy Lifestyle" OR “Quality of life” OR "Quality of Life/psychology" OR “Psychological factors” OR “Funcionality” )) OR MH: (("Shoulder Pain" OR "Shoulder" OR "Tendinopathy" OR "Rotator Cuff Injuries" OR "Bursitis" OR "Synovitis" OR "Shoulder Impingement Syndrome" OR "Rotator Cuff Tear Arthropathy") AND ("Circadian Clocks" OR "Circadian Rhythm" OR "Chronobiology Discipline" OR "Sleep Wake Disorders" OR "insulin" OR "Fasting" OR "Caloric Restriction" OR "Diet, Ketogenic") AND ("Pain" OR "Chronic Pain" OR "Healthy Lifestyle" OR "Quality of Life/psychology")) |
| Web Of Science | TS=(("Shoulder pain” OR "shoulder" OR “Chronic shoulder pain” OR “Tendinopathy” OR “Tenosynovitis” OR “Tendinitis” OR “Tendinosis” OR “Bursitis” OR “Adhesive capsulitis” OR “Frozen shoulder” OR “Synovitis” OR “Rotator cuff” OR "Rotator Cuff Injuries" OR "Rotator Cuff Tear Arthropathy" OR “Shoulder impingement syndrome” OR “Shoulder Arthritis”) AND ("Circadian Clocks" OR "Circadian Rhythm" OR “Chronobiology Discipline" OR "Sleep Wake Disorders" OR "insulin" OR "Fasting" OR "Caloric Restriction" OR "Diet, Ketogenic" OR “Nutritional strategies” OR “Therapy”) AND ("Pain" OR "Chronic Pain" OR "Healthy" OR "Healthy Lifestyle" OR “Quality of life” OR "Quality of Life/psychology" OR “Psychological factors” OR “Funcionality” ) |
| Scopuss | (("Shoulder pain” OR "shoulder" OR “Chronic shoulder pain” OR “Tendinopathy” OR “Tenosynovitis” OR “Tendinitis” OR “Tendinosis” OR “Bursitis” OR “Adhesive capsulitis” OR “Frozen shoulder” OR “Synovitis” OR “Rotator cuff” OR "Rotator Cuff Injuries" OR "Rotator Cuff Tear Arthropathy" OR “Shoulder impingement syndrome” OR “Shoulder Arthritis”) AND ("Circadian Clocks" OR "Circadian Rhythm" OR “Chronobiology Discipline" OR "Sleep Wake Disorders" OR "insulin" OR "Fasting" OR "Caloric Restriction" OR "Diet, Ketogenic" OR “Nutritional strategies” OR “Therapy”) AND ("Pain" OR "Chronic Pain" OR "Healthy" OR "Healthy Lifestyle" OR “Quality of life” OR "Quality of Life/psychology" OR “Psychological factors” OR “Funcionality” )) OR (("Shoulder Pain" OR "Shoulder" OR "Tendinopathy" OR "Rotator Cuff Injuries" OR "Bursitis" OR "Synovitis" OR "Shoulder Impingement Syndrome" OR "Rotator Cuff Tear Arthropathy") AND ("Circadian Clocks" OR "Circadian Rhythm" OR "Chronobiology Discipline" OR "Sleep Wake Disorders" OR "insulin" OR "Fasting" OR "Caloric Restriction" OR "Diet, Ketogenic") AND ("Pain" OR "Chronic Pain" OR "Healthy Lifestyle" OR "Quality of Life/psychology")) |

**Supplementary table 2: Grade System.**


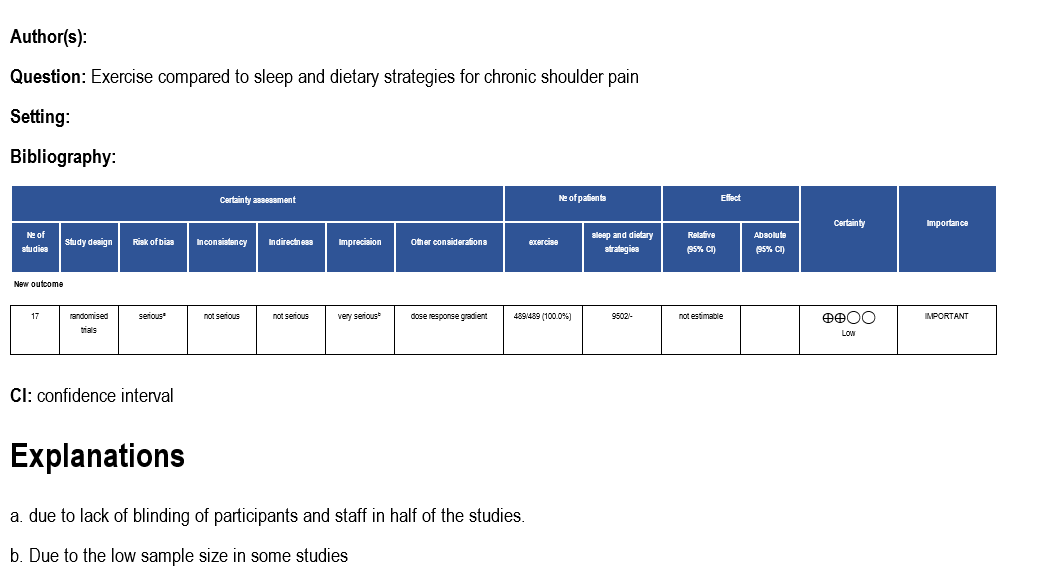

Supplement: Supplementary file 1 [file DataSheet1.docx]
